# Investigating Direct and Indirect Genetic Effects in Attention-Deficit/Hyperactivity Disorder Using Parent-Offspring Trios

**DOI:** 10.1016/j.biopsych.2022.06.008

**Published:** 2023-01-01

**Authors:** Joanna Martin, Matthew Wray, Sharifah Shameem Agha, Katie J.S. Lewis, Richard J.L. Anney, Michael C. O’Donovan, Anita Thapar, Kate Langley

**Affiliations:** a MRC Centre for Neuropsychiatric Genetics and Genomics, Division of Psychological Medicine and Clinical Neurosciences, Cardiff University, Cardiff, United Kingdom; b Wolfson Centre for Young People’s Mental Health, Division of Psychological Medicine and Clinical Neurosciences, Cardiff University, Cardiff, United Kingdom; c School of Psychology, Cardiff University, Cardiff, United Kingdom; d Cwm Taf Morgannwg University Health Board, Wales, United Kingdom

**Keywords:** ADHD, Case-control design, Genetic nurture, Missing data, Polygenic risk scores, Trio design

## Abstract

**Background:**

Attention-deficit/hyperactivity disorder (ADHD) is highly heritable, but little is known about the relative effects of transmitted (i.e., direct) and nontransmitted (i.e., indirect) common variant risks. Using parent-offspring trios, we tested whether polygenic liability for neurodevelopmental and psychiatric disorders and lower cognitive ability is overtransmitted to ADHD probands. We also tested for indirect or genetic nurture effects by examining whether nontransmitted ADHD polygenic liability is elevated. Finally, we examined whether complete trios are representative of the clinical ADHD population.

**Methods:**

Polygenic risk scores (PRSs) for ADHD, anxiety, autism, bipolar disorder, depression, obsessive-compulsive disorder, schizophrenia, Tourette syndrome, and cognitive ability were calculated in UK control subjects (*n* = 5081), UK probands with ADHD (*n* = 857), their biological parents (*n* = 328 trios), and also a replication sample of 844 ADHD trios.

**Results:**

ADHD PRSs were overtransmitted and cognitive ability and obsessive-compulsive disorder PRSs were undertransmitted. These results were independently replicated. Overtransmission of polygenic liability was not observed for other disorders. Nontransmitted alleles were not enriched for ADHD liability compared with control subjects. Probands from incomplete trios had more hyperactive-impulsive and conduct disorder symptoms, lower IQ, and lower socioeconomic status than complete trios. PRS did not vary by trio status.

**Conclusions:**

The results support direct transmission of polygenic liability for ADHD and cognitive ability from parents to offspring, but not for other neurodevelopmental/psychiatric disorders. They also suggest that nontransmitted neurodevelopmental/psychiatric parental alleles do not contribute indirectly to ADHD via genetic nurture. Furthermore, ascertainment of complete ADHD trios may be nonrandom, in terms of demographic and clinical factors.

SEE COMMENTARY ON PAGE 6

Attention-deficit/hyperactivity disorder (ADHD) is a highly heritable neurodevelopmental disorder, with robustly associated common genetic risk variants (1). It shares genetic liability with many other neurodevelopmental/psychiatric disorders, and lower cognitive ability/IQ (1, 2, 3). Parents of children with ADHD have a higher prevalence of ADHD and other neurodevelopmental/psychiatric disorders than the general population (4,5). Given that ADHD is highly heritable, cross-generational transmission is likely explained by genetic, rather than environmental, factors. However, parents provide the pre- and postnatal environment for their children, both of which have an effect on early development. It is well established that many environmental exposures are influenced by parental genotypes; known as gene-environment correlation (6). As such, it is possible that ADHD is influenced by parental genetic liability that is not transmitted to the child, via indirect or genetic nurture effects (e.g., via parenting behavior), in addition to transmitted or direct genetic risks.

ADHD genetic studies frequently use a case-control design (1), but an alternative parent- offspring trio design (using data from an ADHD proband and both biological parents) is more suitable for certain research purposes. For example, trios can be used to identify inherited and noninherited genetic risk variants (7). This design circumvents limitations of the case-control design, such as imperfect case-control matching on confounders (e.g., ancestry) and bias from the use of screened control subjects in cross-disorder genetic correlation estimates (8). More recently, the trio design has been extended to allow the study of transmission of total common variant liability from parents to offspring (9), and to test the impact of indirect genetic effects or genetic nurture on children, by examining the contribution of nontransmitted alleles (10). Enriched ADHD polygenic liability in nontransmitted parental alleles could exert an effect on the proband through such an indirect genetic nurture path.

The first aim of this study was to test whether polygenic risk scores (PRSs) (or the sum of each individual’s common variant liability) for ADHD, other neurodevelopmental/psychiatric disorders, and lower cognitive ability are overtransmitted from parents to children with ADHD (i.e., direct genetic effects). Given the high heritability of ADHD, we expected to observe overtransmission of risk alleles for ADHD and phenotypes with which ADHD shares genetic liability. This can be tested using the polygenic transmission disequilibrium test (pTDT), which compares proband PRS to the mean of their parents’ PRSs (i.e., the common variant liability expected in the proband by chance) (9). Under the hypothesis that manifestation of ADHD depends on direct genetic effects, risk alleles must be transmitted to the proband more often than expected by chance, resulting in a proband PRS greater than the parental mean. When testing for shared cross-disorder genetic effects, this is a more stringent test than case-control analysis, given the limitations of case-control samples outlined above.

The second aim was to investigate whether nontransmitted ADHD risk alleles are elevated in parents of children with ADHD, compared with population control subjects. Genome-wide association studies (GWASs) of trios use pseudo-control alleles, which represent the parental alleles that are not transmitted to offspring. If the parents have ADHD or multiple offspring with ADHD, these nontransmitted alleles could be enriched for ADHD liability compared with the general population, which could reduce genomic discovery power (11,12). Of even greater interest, nontransmitted alleles can exert indirect genetic effects on offspring phenotype (e.g., via the environment parents provide). Enrichment of ADHD risk in nontransmitted alleles compared with control subjects would be consistent with indirect or genetic nurture effects on offspring ADHD. Therefore, a better understanding of the factors influencing ADHD risk can help inform early intervention and prevention strategies. Such indirect effects may also exist for phenotypes that share genetic liability with ADHD (e.g., other neurodevelopmental/psychiatric disorders and lower cognitive ability).

A final question was whether children with ADHD recruited into a trio design are representative of the wider clinical ADHD population. The need to obtain DNA from all 3 individuals in a parent-offspring trio could result in biased ascertainment. This can be a challenge because missing genetic information is unlikely to be missing at random (13). Many children with ADHD do not live with both biological parents, with evidence that ADHD severity is linked to likelihood of a child’s being in a nonintact family (14,15). Previous research by our group suggests that in cases in which fathers do not live with the family, or decline to take part in research, children are more likely to have the more severe DSM-IV combined subtype of ADHD and comorbid conduct disorder (CD) than those from intact families (16). This could mean that probands from incomplete trios have higher genetic liability for ADHD (and related disorders), affecting the generalizability of studies ascertaining only trios, but this requires investigation.

In this study, we tested the following hypotheses using a UK clinical sample of children diagnosed with ADHD and their biological parents: 1) children with ADHD disproportionately inherit liability for neurodevelopmental/psychiatric disorders and lower cognitive ability, 2) nontransmitted ADHD polygenic liability is elevated compared with control subjects (i.e., evidence of genetic nurture), and 3) children from incomplete trios have a more severe clinical profile and higher neurodevelopmental/psychiatric polygenic liability, than those from complete trios.

## Methods and Materials

### Sample Description

Children and young people with ADHD (ages 5–18 years; hereafter referred to as probands) were recruited through child and adolescent psychiatry or pediatric outpatient clinics across Wales and England. Exclusion criteria were a clinical diagnosis of schizophrenia, history of epilepsy, brain damage, or known neurologic or genetic disorder. Inclusion criteria were a DSM-III-R/DSM-IV research-based diagnosis for ADHD, confirmed using the Child and Adolescent Psychiatric Assessment (17), a semistructured diagnostic interview undertaken with parents by trained and supervised psychologists, which assesses DSM-IV inattentive and hyperactive-impulsive symptoms, 2 additional DSM-III-R symptoms, and impairment. Symptom pervasiveness across settings was confirmed using teacher reports [Child ADHD Teacher Telephone Interview (18), or Conners’ Teacher Rating Scale (19)].

Written informed consent was obtained from all parents and young people 16 to 18 years of age and assent was gained from probands <16 years of age. Study approval was obtained from the Northwest England and Wales Multicentre Research Ethics Committees.

Inattentive and hyperactive-impulsive symptom scores were generated using DSM-IV criteria. Impairment was assessed using 8 items (home life, social interactions, community activities, school, sports/clubs, taking care of oneself, recreational activities, and handling responsibilities). Impairment occurring “sometimes” or “often” was coded as 1 and “never” or “rarely” coded as 0, and items were summed.

The Child and Adolescent Psychiatric Assessment was also used to assess comorbid symptoms in the preceding 3 months, according to DSM-IV, including CD, oppositional defiant disorder, anxiety, and depression. Probands 12 years of age and older also completed the child version of the Child and Adolescent Psychiatric Assessment. A symptom was considered as present if either the parent or proband reported it. Total symptom scores for CD (9 items), oppositional defiant disorder (8 items), anxiety (12 items), and depression (8 items) were generated. Autistic traits were assessed using the parent-rated Social Communication Questionnaire (39 items) (20). Full-scale IQ was assessed using the Wechsler Intelligence Scale for Children (WISC), version III/IV (21,22). Probands with IQ < 70 were considered to have intellectual disability (ID).

Socioeconomic variables (family income, parental educational attainment, and parental employment status) and family history of psychiatric disorders were assessed by parental questionnaire. Low income was defined as self-reported gross annual family income <£20,000 (equivalent ∼US$32,000). Parental low educational attainment was defined as parents having left school without qualifications (General Certificate of Secondary Education or equivalent) at age 16 years. Socioeconomic status (SES) was classified by the occupation of the main family wage earner using the UK Standard Occupation Classification (23). Two SES categories were defined (low: unskilled workers/unemployed; medium/high: manual and nonmanual skilled/partially skilled workers and professional/managerial workers). Family history was based on reported information about first degree relatives (i.e., biological parents and full siblings). Three binary variables were derived, relating to the presence of ADHD, other neurodevelopmental problems (e.g., learning difficulties, dyslexia, dyspraxia), and broadly defined major psychiatric disorders (e.g., depression, bipolar disorder [BD], schizophrenia).

### Genetic Data

A detailed description of the genetic data can be found in the Supplement. In brief, DNA samples were collected from probands and parents and genotyped, followed by rigorous quality control (QC) procedures. Parent-offspring relationships were confirmed using identity-by-descent analysis in PLINK. The study ascertained families of European ancestry, which was confirmed using principal components analysis. For complete trios, nontransmitted parental alleles were extracted using PLINK (function: --tucc).

PRSs were calculated using common autosomal variants based on 9 large discovery GWASs of primarily European ancestry: ADHD (1), anxiety disorders (24), autism spectrum disorder (ASD) (25), BD (26), major depressive disorder (MDD) (27), schizophrenia (28), obsessive-compulsive disorder (OCD) (29), Tourette syndrome (30), and cognitive ability/IQ (31).

Comparison individuals were 5081 individuals from the Wellcome Trust Case Control Consortium (WTCCC) (a UK control population sample) not screened for ADHD or other psychiatric disorders (32). The sample has been used previously as control subjects for a GWAS including a subset of the current ADHD cases (1,33). The ADHD sample (including the ADHD nontransmitted parental pseudo-genotypes) was merged with the population control subjects using shared single nucleotide polymorphisms.

The discovery GWAS used to calculate PRS had no overlap with the target ADHD sample. For the merged ADHD-control sample, we obtained GWAS data excluding the control subjects, where possible (all except MDD and BD).

PRSs were calculated using linkage disequilibrium-clumping in PLINK (34) for 7 *p* value thresholds and the first principal component was extracted and analyzed for each discovery phenotype following the PRS-principal components analysis method, an approach that reduces overfitting, maintaining good power (35); see details in the Supplement. PRSs in the merged ADHD-control sample were standardized using the control population mean and standard deviation. Otherwise, PRSs were standardized as *z* scores.

PCAiR (36), a package that robustly estimates population structure while taking into account kinship information, was used to extract the top 10 principal components.

### Definition of Trio Status

A total of 857 probands with ADHD (mean [SD] age = 10.4 [2.8] years; *n* = 119 [13.9%] female) from 825 families met inclusion criteria and passed QC. Complete trios (coded as 0) were defined as families in which both parents provided a DNA sample and were confirmed as the biological parents, regardless of whether both parents passed subsequent QC (*n* = 367). Incomplete trios (coded as 1) were families in which 1 or both parents did not provide a DNA sample (*n* = 454). In families in which both parents had provided DNA but 1 or both parents could not be genotyped because of low sample quality, the probands were unclassified, because parent-offspring relatedness could not be confirmed (*n* = 36).

Additional filters were applied for the analyses of transmitted (pTDT) and nontransmitted alleles, as follows: probands were excluded if parental samples did not pass QC or if the whole trio was not genotyped on the same array. Only the oldest proband was included for families with multiple probands genotyped (*n* = 39 excluded). This resulted in a sample of 328 trios meeting inclusion criteria for the pTDT and the analysis of nontransmitted parental alleles.

### Analyses

We tested for overtransmission of liability to ADHD, ASD, anxiety, BD, MDD, OCD, schizophrenia, and Tourette syndrome and undertransmission of liability to cognitive ability in complete trios using the pTDT (9). This analysis tests whether, on average, the proband PRS deviates significantly from the parental midpoint PRS and is robust to population stratification and other potential confounders (e.g., SES).

Next, we compared the ADHD PRSs in nontransmitted alleles to population control subjects. We also explored whether the PRSs for other neurodevelopmental/psychiatric disorders and cognitive ability differed between nontransmitted alleles and population control subjects.

Finally, we compared probands from complete and incomplete trios, in terms of demographic variables, clinical symptoms, and socioeconomic variables, and the proband and mother’s PRS for ADHD and related phenotypes. Father’s PRS could not be compared because there were only 14 fathers in incomplete trios.

The top 10 ancestry-based principal components were residualized out of the PRS prior to analysis (except for the pTDT). When comparing probands and mothers based on trio status, genotyping batch was also included as a covariate and we accounted for the presence of siblings by specifying family clusters and applying a sandwich estimator to estimate cluster-robust standard errors of regression coefficients. All analyses used generalized estimating equations implemented in the drgee package in R. False discovery rate correction for multiple testing was applied for the genetic analyses in the primary sample.

### Replication Analysis

Independent data from the International Multicentre ADHD Genetics (IMAGE) study (37) were used for replication. The sample consisted of 844 complete trios of probands diagnosed with ADHD (mean [SD] age = 10.9 [2.8] years; *n* = 111 [13.2%] females). Only 616 complete trios matched the WTCCC control population sample ancestry for the analyses of nontransmitted parental alleles. See the Supplement for details. Replication analyses were not corrected for multiple tests because the results are only interpreted with regard to how they compare with the primary analyses.

## Results

### Polygenic Transmission

**Figure 1:**
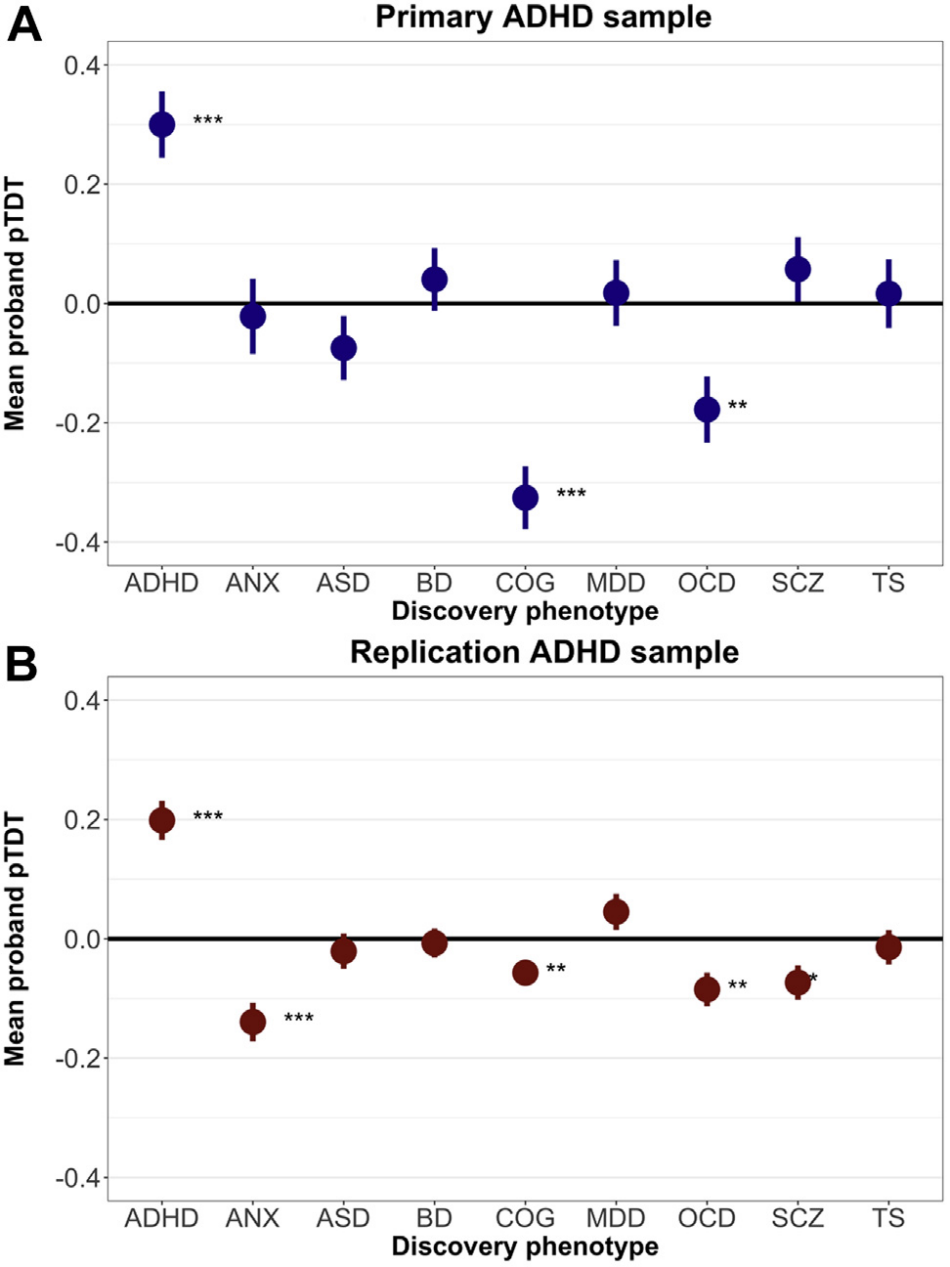
Mean deviation of proband polygenic risk scores from the midparent distribution (i.e., standard deviations away from the midparent distribution) in attention-deficit/hyperactivity disorder (ADHD) trios, using the **(A)** primary sample (*n* = 328 trios) and the **(B)** replication sample (*n* = 844 trios). *p* values indicate the probability that the mean of the polygenic transmission disequilibrium test (pTDT) deviation distribution is 0 (two-sided, 1-sample *t* test). Error bars indicate standard errors. ∗*p* < .05; ∗∗*p* < .01; ∗∗∗*p* < .001. *p* Values shown are corrected for multiple tests for primary analyses and raw *p* values are shown for the replication analyses. See Table S1 for detailed results. ANX, anxiety disorders; ASD, autism spectrum disorder; BD, bipolar disorder; COG, cognitive ability; MDD, major depressive disorder; OCD, obsessive-compulsive disorder; SCZ, schizophrenia; TS, Tourette syndrome.

PRSs for ADHD were overtransmitted (mean [SE] = 0.30 [0.06]) to probands. There was no evidence of overtransmission of risk alleles for other disorders (Figure 1A; Table S1). Polygenic liabilities for cognitive ability (−0.33 [0.05]) and OCD (−0.18 [0.05]) were undertransmitted. The cognitive ability results were not influenced by comorbid ID; after excluding 28 ADHD probands with ID, the results remained the same (−0.33 [0.05]).

The results were independently replicated for ADHD (0.20 [0.03]), cognitive ability (−0.06 [0.02]), and OCD (−0.08 [0.03]) PRSs (Figure 1B; Table S1). Analyses in the replication sample also indicated undertransmission of anxiety and schizophrenia PRSs, but this was not supported by primary analyses.

### Nontransmitted Parental Alleles

**Figure 2:**
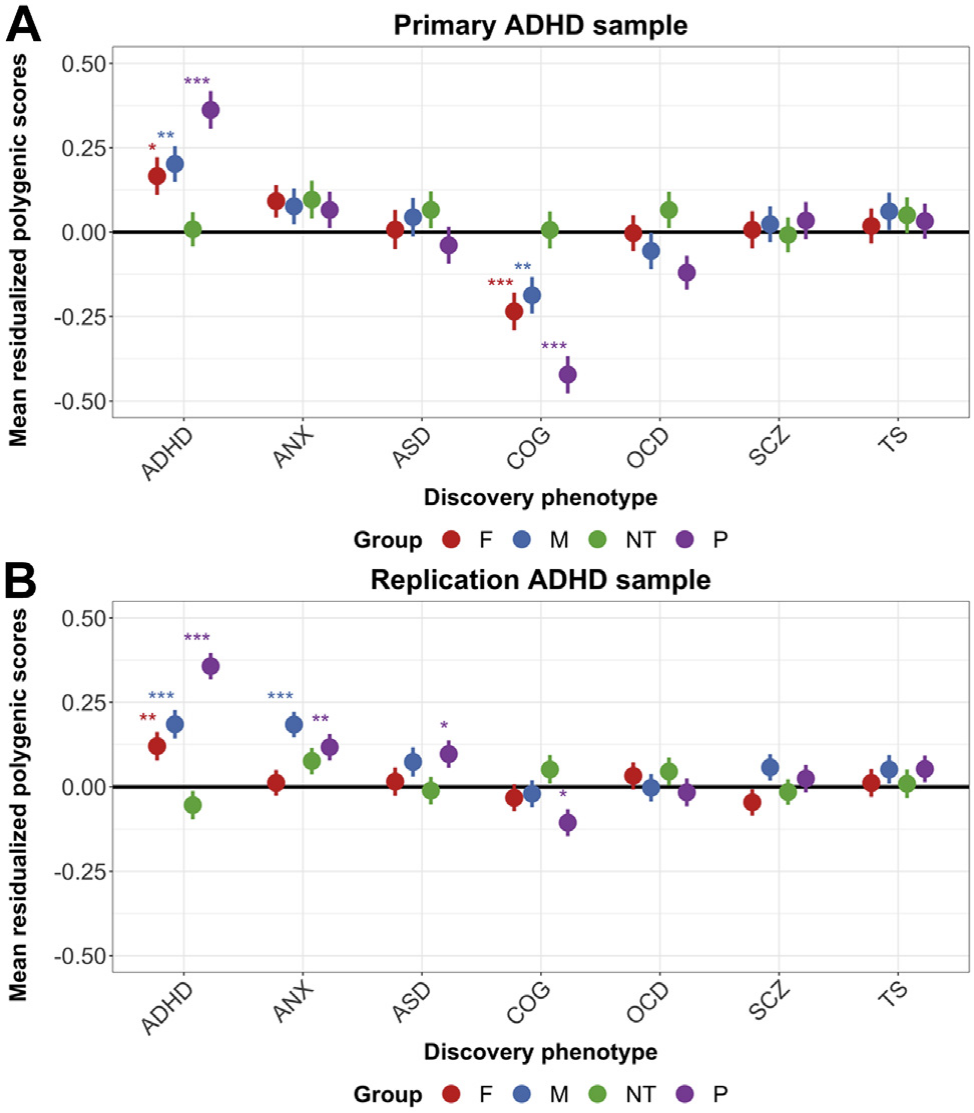
Mean polygenic risk scores in attention-deficit/hyperactivity disorder (ADHD) complete trios: **(A)** primary sample (*n* = 328) and **(B)** replication sample (*n* = 616), in probands (P), fathers (F), mothers (M), and nontransmitted parental alleles (NT), relative to the control population sample (bold horizontal line at *y* = 0). Note: Bipolar disorder and major depressive disorder could not be examined because of inclusion of the control population sample in the discovery genetic studies for those disorders. Error bars indicate standard errors. ∗*p* < .05; ∗∗*p* < .01; ∗∗∗*p* < .001. *p* Values shown are corrected for multiple tests for primary analyses and raw *p* values are shown for the replication analyses. See Table S2 for detailed results. ANX, anxiety disorders; ASD, autism spectrum disorder; COG, cognitive ability; OCD, obsessive-compulsive disorder; SCZ, schizophrenia; TS, Tourette syndrome.

Figure 2A displays the mean PRSs for 328 ADHD trios, separately for probands, mothers, fathers, and nontransmitted parental alleles, relative to the control population; see Table S2 for detailed results. There was no evidence supporting elevated ADHD PRS for nontransmitted parental alleles. Exploratory analyses also found little support for elevated nontransmitted parental PRS for other disorders or lower cognitive ability PRSs. PRSs of probands, fathers, and mothers were elevated for ADHD and lower for cognitive ability than control subjects. No significant differences were observed for other disorder PRSs.

Analyses in the replication sample are shown in Figure 2B and Table S2. The results of the primary analysis were replicated for ADHD PRSs. Cognitive ability PRSs were lower and ASD PRSs were elevated in probands compared with control subjects. Proband and mothers’ anxiety PRSs were elevated compared with control subjects. There was little evidence of elevated PRSs for nontransmitted parental alleles for any phenotypes compared with the control sample and no other group differences were observed.

### Complete and Incomplete Trios

Analysis of 821 probands (complete trios: *n* = 367, incomplete trios: *n* = 454) indicated that those from incomplete trios were older, had more hyperactive-impulsive ADHD and CD symptoms, had lower IQ, and were more likely to meet diagnostic criteria for CD (Table 1). Other variables were similar between groups. Parents of probands from incomplete trios had lower educational attainment, annual family income, and SES based on occupation. There were no group differences in family history of neurodevelopmental/psychiatric disorders.

**Table 1:** Comparison of Probands From Complete (*n* = 367) and Incomplete (*n* = 454) Trios in Terms of Demographic, Clinical, Socioeconomic, and Family History Variables

Phenotype	Incomplete Trios, Mean (SE) or *n* (%)	Complete Trios, Mean (SE) or *n* (%)	OR (95% CI)^a^	*p*
Proband Age^b^	10.70 (0.13)	10.10 (0.15)	1.07 (1.02–1.13)	6.3 × 10^−3^^c^
Inattentive Symptoms	7.48 (0.08)	7.27 (0.09)	1.07 (0.98–1.17)	.11
Hyperactive-Impulsive Symptoms	7.82 (0.07)	7.65 (0.08)	1.12 (1.02–1.24)	.019^c^
ADHD Impairment	6.88 (0.07)	6.69 (0.09)	1.11 (0.99–1.24)	.087
IQ	82.90 (0.67)	85.50 (0.71)	0.99 (0.98–1.00)	.041^c^
Autistic Traits	13.50 (0.36)	12.80 (0.46)	1.02 (0.99–1.04)	.23
Anxiety Symptoms	1.02 (0.09)	0.93 (0.10)	1.03 (0.93–1.13)	.60
Depressive Symptoms	1.42 (0.07)	1.21 (0.07)	1.09 (0.98–1.22)	.11
ODD Symptoms	4.11 (0.11)	3.90 (0.13)	1.04 (0.98–1.10)	.19
CD Symptoms	1.53 (0.08)	1.16 (0.09)	1.12 (1.02–1.23)	.012^c^
Proband Sex, Male	72 (15.9%)	46 (12.5%)	1.35 (0.91–2.01)	.13
Low Family Income	212 (72.4%)	105 (50.5%)	2.64 (1.78–3.90)	1.3 × 10^−6^^c^
Low Parental Education	100 (31.6%)	47 (21.9%)	1.67 (1.10–2.52)	.015
Low Family SES	230 (59.9%)	125 (39.1%)	2.35 (1.71–3.22)	1.1 × 10^−7^^c^
Intellectual Disability	58 (13.6%)	29 (8.2%)	1.27 (1.00–1.61)	.055
CD Diagnosis	107 (23.7%)	60 (16.4%)	1.51 (1.05–2.18)	.025^c^
Family History				
ADHD	75 (23.7%)	60 (25.0%)	0.92 (0.60–1.42)	.71
Other NDs	40 (12.9%)	32 (13.4%)	0.95 (0.56–1.60)	.84
Major psychiatric disorders	139 (42.4%)	89 (37.4%)	1.24 (0.87–1.77)	.24

ADHD, attention-deficit/hyperactivity disorder; CD, conduct disorder; NDs, neurodevelopmental disorders; ODD, oppositional defiant disorder; OR, odds ratio; SES, socioeconomic status.

a Probands from complete trios were coded as 0 and those from incomplete trios were coded as 1; therefore, OR > 1 can be interpreted as an increase of the variable in the probands from incomplete trios.

b Proband age is included as a covariate in all other analyses.

c *p* < .05.

Analysis of probands’ and mothers’ PRSs indicated several nominally significant effects (Table 2). Probands from incomplete trios had higher BD and OCD PRSs, and higher maternal ADHD PRSs. On the contrary, mothers’ schizophrenia PRSs were lower in incomplete trios. However, these effects did not withstand multiple testing correction.

**Table 2:** Comparison of Polygenic Risk Scores in Probands and Mothers From Complete (*n* = 367)^a^ and Incomplete (*n* = 454) Trios

PRS	OR (95% CIs)	*p*	*p*_FDR_
Proband’s PRS			
ADHD	1.31 (0.94–1.82)	.12	.27
ANX	1.11 (0.79–1.54)	.55	.62
ASD	0.86 (0.62–1.17)	.33	.54
BD	1.32 (1.03–1.69)	.026	.21
COG	0.86 (0.58–1.26)	.43	.58
MDD	1.38 (0.87–2.18)	.17	.34
OCD	1.35 (1.00–1.81)	.047	.21
SCZ	0.93 (0.70–1.22)	.60	.64
TS	1.29 (0.98–1.70)	.073	.23
Mother’s PRS			
ADHD	1.59 (1.06–2.40)	.026	.21
ANX	1.26 (0.74–2.15)	.40	.58
ASD	1.30 (0.62–2.72)	.48	.58
BD	0.72 (0.50–1.04)	.078	.23
COG	1.08 (0.64–1.82)	.77	.77
MDD	1.58 (0.80–3.15)	.19	.34
OCD	1.21 (0.73–2.00)	.45	.58
SCZ	0.59 (0.36–0.97)	.038	.21
TS	1.37 (0.95–1.98)	.097	.25

ADHD, attention-deficit/hyperactivity disorder; ANX, anxiety disorders; ASD, autism spectrum disorder; BD, bipolar disorder; COG, cognitive ability; FDR, false discovery rate; MDD, major depressive disorder; OCD, obsessive-compulsive disorder; OR, odds ratio; PRS, polygenic risk score; SCZ, schizophrenia; TS, Tourette syndrome.

a Genetic data were available for 344 mothers from complete trios and 269 mothers from incomplete trios.

## Discussion

We used a parent-offspring ADHD trio sample to test 3 hypotheses: overtransmission of polygenic liability from parents to probands, elevated polygenic liability in nontransmitted parental alleles, and nonrepresentativeness of ADHD trios. We found robust evidence of overtransmission of ADHD and lower cognitive ability polygenic liability and evidence of undertransmission of OCD PRSs. This was replicated in an independent ADHD sample and consistent with case-control analysis. We found limited evidence of overtransmission or case-control differences for other disorder PRSs. Parental nontransmitted alleles related to ADHD and other phenotypes were not elevated compared with a control population; i.e., we observed no evidence of genetic nurture. Finally, we observed several clinical and socioeconomic differences between probands from complete and incomplete trios, but no robust differences in PRSs.

Overtransmission of ADHD polygenic liability, while not previously tested in ADHD using the pTDT, was expected. Interestingly, the magnitude of the effect sizes observed for ADHD and cognitive ability were similar in the primary sample, despite differences in size and genetic architecture of these discovery GWASs. ADHD is strongly associated with lower cognitive ability, and twin studies have shown a genetic correlation of 91% between ADHD and ID (38). Excluding 28 probands with ID did not affect the results. These results were replicated in an independent and larger ADHD sample from various European countries, although the effect sizes were smaller, particularly for cognitive ability. The primary sample’s IQ was lower than the population average (mean [SE] = 84.2 [0.48]), as expected for ADHD (39). The mean IQ of the replication sample was comparable to the population average (100.4 [0.66]), and only 10 probands had IQ of <70, which could explain the lower effect size observed. However, the replicated result suggests that overtransmission of lower cognitive ability polygenic liability is not entirely explained by individuals with lower IQ.

Previous estimates of genetic correlation between ADHD and cognitive ability (*r*_g_ = −0.41) are of a similar magnitude to those between ADHD and MDD (*r*_g_ = 0.42), ASD (*r*_g_ = 0.36), and anxiety (*r*_g_ = 0.33) (1,24,25,27). Despite these similar genetic correlations, we do not see evidence of overtransmission of PRS or case-control differences for these other disorders in our study. It is possible that genetic correlations estimated in previous studies have been overestimated, for example by inclusion of comorbid cases in discovery GWASs (e.g., individuals with both ADHD and MDD in GWASs of each disorder) or through use of overscreened control subjects (8). It is also likely that differences in genetic architecture across phenotypes (e.g., smaller total contribution of common variants to heritability for MDD and other disorders) affected these results and that larger sample sizes are needed to detect shared genetic effects. Alternatively, it could be that probands with ADHD who overinherit polygenic liability for both ADHD and other disorders from their parents may show a different phenotype (e.g., ADHD and comorbid BD or psychosis) and meet study exclusion criteria or are less likely to take part in genetic studies and were thus missing from our sample. The replicated undertransmission of OCD PRSs is consistent with recently reported negative genetic correlations (*r*_g_ [SE] = −0.17 [0.07]) between ADHD and OCD (40) and needs further consideration in future studies. It is possible that probands with a comorbid presentation of ADHD and OCD were less likely to have been included as trios in the current study. This possibility is supported by the slightly higher observed OCD PRSs in the incomplete trios, albeit this result did not survive correction for multiple testing. It has also been suggested that ADHD and OCD represent opposite extremes of the impulsivity-compulsivity continuum, which could explain the opposite directions of genetic effects (40).

Contrary to the second hypothesis we tested, we found no evidence in either sample of elevated polygenic liability for ADHD in the nontransmitted parental alleles, compared with a control population. Similarly, the results of our exploratory analyses indicated no enrichment of nontransmitted parental alleles for polygenic liability for other neurodevelopmental/psychiatric disorders or for lower cognitive ability. These results are consistent with a recent study and also a preprint, which examined ADHD symptoms in the general population (41,42). Our study is different because it focuses on clinical ADHD diagnosis. These studies found that the nontransmitted parental alleles for ADHD and educational attainment do not contribute to risk of ADHD symptoms in a general childhood population, in contrast to transmitted parental alleles (41,42). Together with our results and the limited role of shared environmental risks in ADHD reported in twin studies (43), this indicates that parental polygenic liability for ADHD primarily impacts on child ADHD risk via direct genetic transmission, rather than indirect genetic nurture effects.

Our results also indicate that common variant discovery GWASs of ADHD may not be adversely affected by the use of pseudo-control subjects from trios relative to case-control samples, as has been previously suggested (11). It is likely that nontransmitted parental risk alleles could be enriched in a subgroup (e.g., families with multiple affected children) or that our use of an unscreened population sample (making this a conservative test) affected the results. This needs to be investigated in future studies. Future studies should also consider the possible effect of comorbid neurodevelopmental/psychiatric symptoms when examining whether nontransmitted risks for other neurodevelopmental/psychiatric phenotypes are enriched in children with ADHD.

Comparison of polygenic liability in complete and incomplete trios indicated weak differences; probands from incomplete trios had elevated PRSs for BD and OCD and their mothers had elevated ADHD PRSs but decreased schizophrenia PRSs. However, these results did not withstand multiple testing correction and require follow-up using larger samples. We were not able to replicate these analyses because incomplete trios were screened out of the IMAGE cohort. There were no differences in family history of ADHD and other disorders. These results indicate that there are no substantial genetic differences (in terms of PRSs or family history) in probands and their mothers depending on whether they were recruited to the study as part of a complete trio or not. As such, ADHD trio samples are reasonably representative of clinical ADHD samples in terms of polygenic background and our results examining polygenic overtransmission and nontransmitted parental alleles reflect typical UK clinical populations.

We observed several demographic and clinical differences depending on trio status. Building on a previous study using a subset of 241 (28%) probands drawn from the current sample (16), we observed that probands from incomplete trios had a more severe clinical profile, with more hyperactive-impulsive ADHD and CD symptoms and lower IQ. Probands from incomplete trios were more likely to meet diagnostic criteria for CD, as previously reported (16). We found no differences in inattentive symptoms, ADHD impairment, or symptoms of oppositional defiant disorder, anxiety, or depression. Thus, the group of probands from incomplete trios were not generally more impaired but rather showed specific differences in clinical profile, relating to cognitive ability and behavioral symptoms. However, we note that some of these group differences showed only small effect sizes. We also found differences in socioeconomic variables, which may be explained by the lower SES of single-parent families (44), who constitute a subset of the incomplete trios. We defined trio status based on availability of DNA from both biological parents, compared with the previous definition of whether fathers of probands with ADHD live with the family and take part in research (16). Although these definitions will overlap, our study is more specifically relevant to considerations of whether trio samples in genetic studies are representative of an ADHD clinical sample and our results indicate that this is not entirely the case.

Our primary target sample was relatively small, which limited our power to detect smaller effects, particularly for indirect genetic effects, which are likely to be smaller for ADHD than the direct genetic effects. However, we can be more confident in the results owing to our use of a comparable replication sample. One limitation of the replication analysis is that we used the same control individuals as in the primary analysis of nontransmitted alleles, which may have influenced the similarity of these results. We were unable to compare fathers’ polygenic profiles given few fathers in incomplete trios. We were also unable to compare nontransmitted parental alleles for MDD and BD with the control population, owing to the inclusion of the control subjects in those discovery GWASs. Although we found only weak evidence of differences in polygenic liability in complete and incomplete trios, this may have affected the analysis of nontransmitted parental alleles, which needs to be studied further.

Overall, our results suggest that probands with ADHD overinherit polygenic liability not just for ADHD but also for lower cognitive ability. We found no evidence of enrichment of polygenic liability for neurodevelopmental or psychiatric phenotypes in nontransmitted parental alleles, suggesting that genetically influenced nurture (as captured by the PRSs we tested) does not contribute to ADHD risk. Finally, our results indicate that probands who are recruited to trio-based genetic study designs may not be entirely representative of clinical samples, showing a somewhat less severe clinical profile and higher family SES.
